# Decoding the Interactions of SM Proteins with SNAREs

**DOI:** 10.1100/tsw.2005.53

**Published:** 2005-06-08

**Authors:** Ren-Wang Peng

**Affiliations:** Max Planck Institute for Biophysical Chemistry, Department of Molecular Genetics, Am Fassberg 11, 37077 Göttingen, Germany

**Keywords:** protein and membrane trafficking, SM proteins, Sly1p, SNAREs, syntaxin, nonsyntaxin SNAREs, Ypt/Rab GTPases

## Abstract

The success and efficiency of fusion of transport vesicles to target membranes depend upon sets of proteins that are functionally and evolutionarily conserved. The soluble *N*-ethylmaleimide-sensitive fusion (NSF)-attachment protein receptor (SNARE) family and the Sec1/Munc18 family, also termed SM proteins, are central players in this process. SM proteins interact with syntaxins of SNARE family, which has been regarded intrinsic for function. By exploring the bi-molecular interactions of SM proteins with syntaxins and their functional implications *in vivo* in several eukaryotes, which has been enormously facilitated by the availability of the three-dimensional structures of members of both protein families, the long-standing assumption that SM proteins fulfill their regulatory role by binding to syntaxins has to be reconsidered.

## INTRODUCTION

Protein and membrane transport in eukaryotic cells is of fundamental importance for exo- and endocytosis and for maintaining the integrity of intracellular organelles. This pathway is mediated by membrane-bound transport vesicles formed on and peeled off a donor compartment where secretory and biosynthetic proteins (cargo) are loaded. The cargo-filled vesicles are then delivered to the cognate destination, resulting in cargo release and integration of vesicular membranes into an acceptor compartment. A key step for this pathway is membrane fusion, the mixing of two phospholipid bilayers of vesicular carriers and target membranes. Membrane fusion consists of several substeps catalyzed by dozens of proteins and protein complexes functioning in coordinated networks. A fundamental question concerning the mechanism of membrane fusion is how these proteins contribute to this step and how their functions are coordinated. In this review, I will focus on Sec1/Munc18 (SM) proteins and their interaction with syntaxin-like SNAREs, and discuss current models of how SM proteins work to regulate membrane fusion.

## SM PROTEINS ARE CORE CONSTITUENT OF MEMBRANE FUSION MACHINERY

A large number of proteins have been characterized and assigned a function in membrane fusion reactions. The evolutionarily conserved families of Rab/Ypt GTPases, Sec1/Munc18 (SM) proteins, and SNARE (soluble *N*-ethylmaleimide-sensitive fusion (NSF)–attachment protein receptor) proteins constitute the core of fusion machinery. According to current knowledge, Rab/Ypt GTPases define the initial recognition (known as "tethering") of transport vesicles to target membranes. SNARE proteins, which bring together the membranes to be fused by forming tight complexes between two opposing lipid bilayers (*trans*-SNARE complexes), apparently drive membrane fusion. But how the equally essential SM proteins operate in this process remains unclear[1,2,3].

The founding members of the SM protein family are Unc-18 of *Caenorhabditis elegans* and Sec1p of *Saccharomyces cerevisiae*, which were identified in genetic screens for uncoordinated phenotypes and mutants in the secretory pathway, respectively[4,5]. Homologs of Sec1p and Unc-18 proteins have since been found in a variety of eukaroytic cells including yeast (Sly1p, Sec1p, Vps45p, and Vps33p), *C. elegans* (Unc-18), *Drosophila* (ROP), and mammals (Munc18 or nSec1), indicating this family is conserved across species[1,2,6,7]. SM proteins identified so far are hydrophilic proteins with molecular mass of 70–90 kDa. The structures of two distantly related SM proteins, mammalian Munc18 and yeast Sly1p, are very similar[8,9], suggesting a conserved function for members of this family. Genetic studies have established a general requirement for SM proteins in membrane fusion of exo- and endocytosis. For example, yeast Sly1p is required for the endoplasmic reticulum (ER)-to-Golgi transport[10,11], and Vps45p and Vps33p for transport between the TGN and endosomes and for homotypic vacuole fusion[12,13,14]. Yeast Sec1p acts in a late step of the secretory pathway[15] and its mammalian counterpart Munc18 has a role in synaptic vesicle exocytosis[16]. It has also been appreciated that the function of SM proteins, like that of SNAREs, is of utmost importance for membrane fusion. This has been clearly illuminated by the fact that loss-of-function mutations in SM proteins lead to abolition of membrane fusion at respective transport routes. For instance, depletion of or conditionally lethal mutations in Sly1p block ER-to-Golgi transport completely[11]. *sec1* mutants accumulate secretory vesicles and are conditionally lethal[17]. Similarly, neurotransmission is reduced in both *C. elegans unc-18*[18] and *Drosophila rop* mutants[19], and both spontaneous and evoked neurotransmitter release are completely blocked in Munc18 knockout mice[20]. These observations argue for an absolute requirement for SM proteins in membrane fusion.

## SM PROTEINS INTERACT WITH SYNTAXINS OF THE SNARE PROTEINS

Many, if not all, SM proteins bind to syntaxins of the SNARE protein family[1]. The most extensively studied example is mammalian Munc18 and syntaxin 1A, two proteins essential for synaptic neurotransmitter release and binding to each other with nanomolar affinity[21]. More detailed studies revealed that syntaxin 1A fluctuates between an open and a closed conformation[22], and Munc18 exclusively binds to the closed syntaxin, in which the N-terminus of syntaxin folds over the C-terminal SNARE motif, thus occluding interactions with other SNARE proteins[8,23]. This interaction involves extensive surface contact of two proteins and requires almost the entire Munc18 and syntaxin molecules[8]. The yeast homologs of Munc18 and syntaxin 1 are Sec1p and Sso1p. Sso1p, like syntaxin 1, adopts open and closed conformations[24]. However, in sharp contrast to Munc18, Sec1p does not bind, or binds very weakly, to isolated Sso1p alone[25]. Instead it prefers the assembled exocytic core complex consisting of Sso1p, Sec9p, and Snc1p[15]. Other examples similar to yeast Sec1p include Sly1p and Vps45p. Sly1p and Vps45p directly bind to their cognate syntaxin homologs[26,27]. Intriguingly, the binding region of Sly1p and Vps45p is limited to the very N-terminus of Sed5p and Tlg2p, respectively[9,28,29]. Importantly, Sly1p binding does not preclude Sed5p from engagement with other SNAREs and instead promotes the formation of physiologically relevant SNARE complexes[30]. Finally, in vacuolar fusion reactions, a large complex known as HOPS, of which Vps33p is a component, but not Vps33p alone, binds to the cognate syntaxin Vam3p, indicating an indirect nature of the binding[31].

In summary, SM proteins interact with syntaxins that act at the same transport routes, but the binding mechanism is not at all uniform. (For illustration, see Fig. 1.)

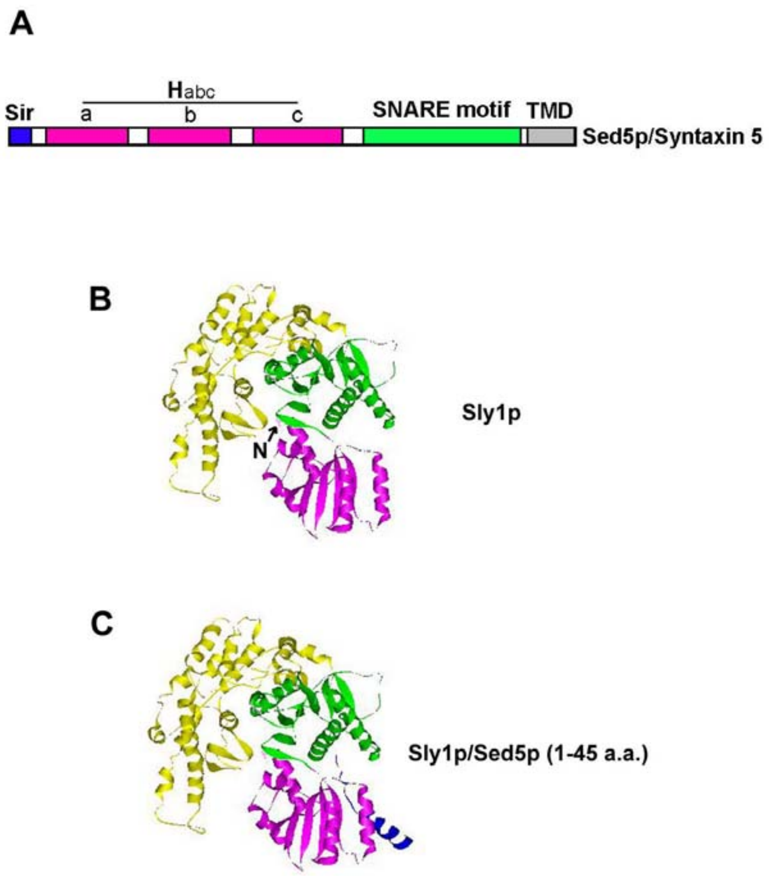

**FIGURE 1.** The interaction of the Golgi syntaxin 5 (Sed5p) and its cognate SM protein Sly1p. (A) Yeast Sed5p and its mammalian homolog syntaxin 5 are characterized by the SNARE motif at the C-terminal proximity and the α-helix a, b, and c, collectively termed Habc domain at the N-terminus. The Sly1p-interacting region (Sir), a stretch of around 20 residues, is located at the very tip of the N-terminal Sed5p. TMD, transmembrane domain. (B) Structure of Sly1p. Sly1p folds into a three-domained (I, II, and III, decorated in red, green, and yellow, respectively), arch-shaped assembly. (C) Structure of Sly1p in complex with the N-terminal 45 amino acids of Sed5p. Sed5p (blue) directly contacts with domain I of Sly1p. The binding region for nonsyntaxin SNAREs is different from that for Sed5p, but it has not been mapped on Sly1p yet. Fig. 1B and 1C were generated from pdb data using ViewerLite 4.2 (http://www.accelrys.com).

## FUNCTIONAL IMPLICATIONS FOR THE INTERACTIONS OF SM PROTEINS WITH SYNTAXINS

The fact that SM proteins interact with syntaxins suggests that the conserved role of SM proteins in membrane fusion might be fulfilled through syntaxins. One possibility is that SM proteins are chaperones for syntaxins. Indeed, yeast Tlg2p becomes unstable when its cognate SM protein Vps45p is mutated[32] and the inhibition of neurotransmitter release in *Drosophila* caused by overexpression of ROP is rescued by concomitantly elevated levels of syntaxin[33]. Also consistent with this idea is that in cell culture, syntaxin is delivered to the plasma membrane only when Munc18 is present[34], and in Munc18 knockout mice, syntaxin levels are reduced by 50%[20,35]. However, as discussed above, not all SM proteins directly bind to syntaxins and the Sed5 protein level remains constant when Sly1p is depleted[30]. It has also been proposed that SM protein binding negatively regulates syntaxin activity[21]. This model apparently cannot be easily reconciled with the fact that deletion of SM proteins abolishes rather than enhances membrane fusion. The third model proposes that the closed conformation of syntaxin is a prerequisite for the opening of syntaxin and for Munc18 binding to facilitate core SNARE complex assembly. However, it is now clear that not all syntaxins adopt an open and a closed conformation and, in many cases, SM proteins bind to both individual syntaxins and syntaxin-containing SNARE complexes[15,30,31]. More importantly, in *C. elegans*, a constitutively open syntaxin fails to bypass the function of the cognate Unc-18, suggesting that SM protein binding is not required for syntaxin opening, which the model would predict[36].

The crystal structures of mammalian Munc18 in complex with syntaxin 1A and yeast Sly1p bound to a short peptide of Sed5p have recently been solved[8,9]. These structural data provide atomic insight into the spatial organization of SM proteins and the bound syntaxins, and therefore are of particular value to test directly the physiological significance of the interaction of the two proteins. The first of such studies was made in *C. elegans*. A null mutation of syntaxin 1 is rescued by a mutant that is constitutively locked in the open conformation. This syntaxin mutant has lost, or has remarkably reduced binding affinity for, its cognate SM protein Unc-18. This strongly suggests that high affinity binding of Unc-18 is dispensable for syntaxin function and for synaptic vesicle exocytosis[37]. A similar finding is obtained from a study in yeast. Sly1p binds to Sed5p as tightly as as Munc18 to syntaxin 1A[26] and structural data revealed that the tight binding is mediated by a hydrophobic pocket of five amino acids in Sly1p surrounding a critical phenylalanine of Sed5p[9]. Mutations in either the hydrophobic pocket of Sly1p or of the phenylalanine of Sed5p abolish the binding of the two proteins *in vitro* and *in vivo*[38]. However, when introduced into yeast, neither the mutant Sly1 nor mutant Sed5 protein causes a growth defect or blocks ER-to-Golgi transport, indicating that the high affinity interaction of Sly1p to Sed5p is not relevant for the functioning of the two proteins[38]. Further, the identification of several Munc18 mutant proteins that affect exocytosis in chromaffin cells, but not the syntaxin binding capacity, also demonstrates a syntaxin-independent function of this SM protein[39].

## SLY1 PROTEIN BINDS TO NONSYNTAXIN SNARES

Sly1p contributes to the pairing of physiologically relevant SNARE complexes[30,40], however, mutant Sly1 proteins unable to bind to Sed5p still function *in vivo*[38]. This suggests that Sly1p has a syntaxin-independent role in regulating core complex assembly. In support of this, Sly1p directly binds to Bet1p and Bos1p, two nonsyntaxin SNAREs of the ER-to-Golgi transport machinery, and to the nonsyntaxin SNAREs Gos1p and Sft1p, whose functions are required for intra-Golgi transport. The Sly1p binding to nonsyntaxin SNAREs is selective, since Sec22p and Ykt6p, the other two nonsyntaxin SNAREs involved in ER-to-Golgi and in intra-Golgi transport routes, respectively, do not bind to Sly1p[38]. Furthermore, a previously unrecognized function of Sly1p in retrograde Golgi-to-ER transport has recently been identified[41]. In agreement with a role in retrograde transport, Sly1p specifically binds to the nonsyntaxin SNAREs Sec20p and Use1p, which are directly required for retrograde Golgi-to-ER transport. Whether the interaction with nonsyntaxin SNAREs is conserved among members of the SM family and how significant the interaction is for SM protein function are awaiting further analyses (see Figure 1).

## CONCLUSIONS AND PERSPECTIVES

As discussed above, SM proteins share a common feature to interact, directly or indirectly, with syntaxins, but the mechanisms underlying the interactions differ remarkably. Given the fact that SM proteins are required for all types of intracellular membrane trafficking, and taking into account the conservation of SM protein structures, it is unlikely that the primary role of SM proteins in membrane fusion is encoded by the interaction with syntaxins, at least is not solely encoded by the interactions.

What kind of protein-protein interaction(s) might account for the essential role of members of this family? At present, different lines of evidence suggests that SM proteins might couple Ypt/Rab GTPase-dependent tethering to SNARE assembly. The best evidence supporting this model comes from studies with yeast homotypic vacuole membrane fusion. The Vps33p-containing HOPS complex directly binds to the GTP-bound Ypt7p, and in the absence of HOPS, the downstream *trans*-SNARE complex fails to assemble. This indicates that HOPS might link tethering to SNARE complex assembly[13,14]. In agreement with this, a single mutation in Sly1p (termed Sly1-20p) suppresses the deletion of Ypt1p[10] and Sly1p physically binds to a selective number of nonsyntaxin SNARE proteins of ER-to-Golgi and intra-Golgi transport, providing a mechanism for SM protein contribution to SNARE assembly[38]. Furthermore, in chromaffin cells, docking of dense core vesicles is impaired in Munc18 knockout mice[35] and in *C. elegans*, *unc-18* mutants strikingly, but specifically, reduce docking of vesicles of the readily releasable pool[36]. This suggests a role for SM proteins prior to SNARE assembly. The characterization of the cascade of protein-protein interactions that control these fusion events will be of crucial importance to understand fully how SM proteins function and how membrane fusion proceeds.

## ACKNOWLEDGMENTS

The author would like to thank Dr. Dieter Gallwitz for suggestions and critical reading of the manuscript, Dr. Hans Dieter Schmitt and Yuejie Li for discussions, and Peter Mienkus for technical assistance.

## BIOSKETCH

**Ren-Wang Peng** received his Ph.D. degree in plant science from the Chinese Academy of Sciences. Now he is a Group Leader at the Department of Molecular Genetics, Max Planck Institute for Biophysical Chemistry, Göttingen, Germany. He studies the molecular mechanism of membrane trafficking that directs the movement of proteins and membranes inside of cells.
